# Case Report: Syncope in an 11-year-old girl induced by anomalous aortic origin of the coronary artery, initially diagnosed via echocardiography

**DOI:** 10.3389/fcvm.2026.1632958

**Published:** 2026-02-18

**Authors:** Juan Wang, Wenlong Wang, Xiaomei Li, Yuting Wu, Xiangqun Sun, Qian Liu, Dong Wang

**Affiliations:** 1 Department of Cardiology, Binzhou Medical University Hospital, Binzhou, Shandong, China; 2 Department of Cardiology, Yantai Affiliated Hospital of Binzhou Medical University, Yantai, Shandong, China; 3 Department of Traditional Chinese Medicine, Binzhou Medical University Hospital, Binzhou, Shandong, China

**Keywords:** anomalous aortic origin of the coronary artery, cardiovascular diseases, case report, coronary vessel anomalies, echocardiography, syncope

## Abstract

Anomalous aortic origin of the coronary artery (AAOCA) is a relatively rare congenital coronary anomaly identified as a common cause of exercise-induced cardiac syncope and sudden death in young individuals. In most cases, the coronary artery courses between the aorta and pulmonary artery, exhibiting an intramural trajectory within the aortic wall. Herein, we present a case of an 11-year-old girl with AAOCA manifesting with sudden-onset syncope complicated by myocardial infarction as the initial symptom, along with a discussion of the underlying pathogenesis. This case is important as it highlights the fact that comprehensive coronary artery evaluation combined with high-quality imaging modalities is critical to enhance the diagnostic accuracy of coronary anomalies. As such, TTE should be established as an important first-line tool within a multimodality imaging pathway. for AAOCA.

## 1 Introduction

Anomalous aortic origin of the coronary artery (AAOCA) is a relatively rare congenital coronary anomaly, representing one of the most common causes of exercise-induced syncope and sudden cardiac death in young individuals (1, 2). This condition is predominantly observed to have an intramural coronary course, where the anomalous artery traverses between the aorta and pulmonary artery while running within the aortic wall (3–5). The pathogenesis of AAOCA primarily involves an intramural aortic course. The path of the coronary artery within the aortic wall, combined with the slit-like ostium and acute-angle takeoff near the left-right coronary sinus junction, leads to reduced coronary blood flow during exertion due to increased intramural wall tension (6–8). During exercise, the coronary artery is compressed by the dilated aorta, exacerbating luminal stenosis or occlusion, and precipitating acute myocardial ischemia, infarction, cardiogenic shock, or syncope (9).

Here, we report a unique case of syncope caused by anomalous origin of (AAOCA), which was initially diagnosed by echocardiography. A standardized echocardiography protocol may improve the diagnostic sensitivity for AAOCA. Transthoracic echocardiography (TTE) should serve as an important first-line tool within a multimodality imaging pathway for AAOCA to enable early diagnosis and reduce the risk of sudden cardiac death. While this article highlights the crucial role of TTE, it must be acknowledged that the gold standard for anatomical definition in AAOCA remains coronary CT angiography.

## 2 Case report

**Figure 1:**
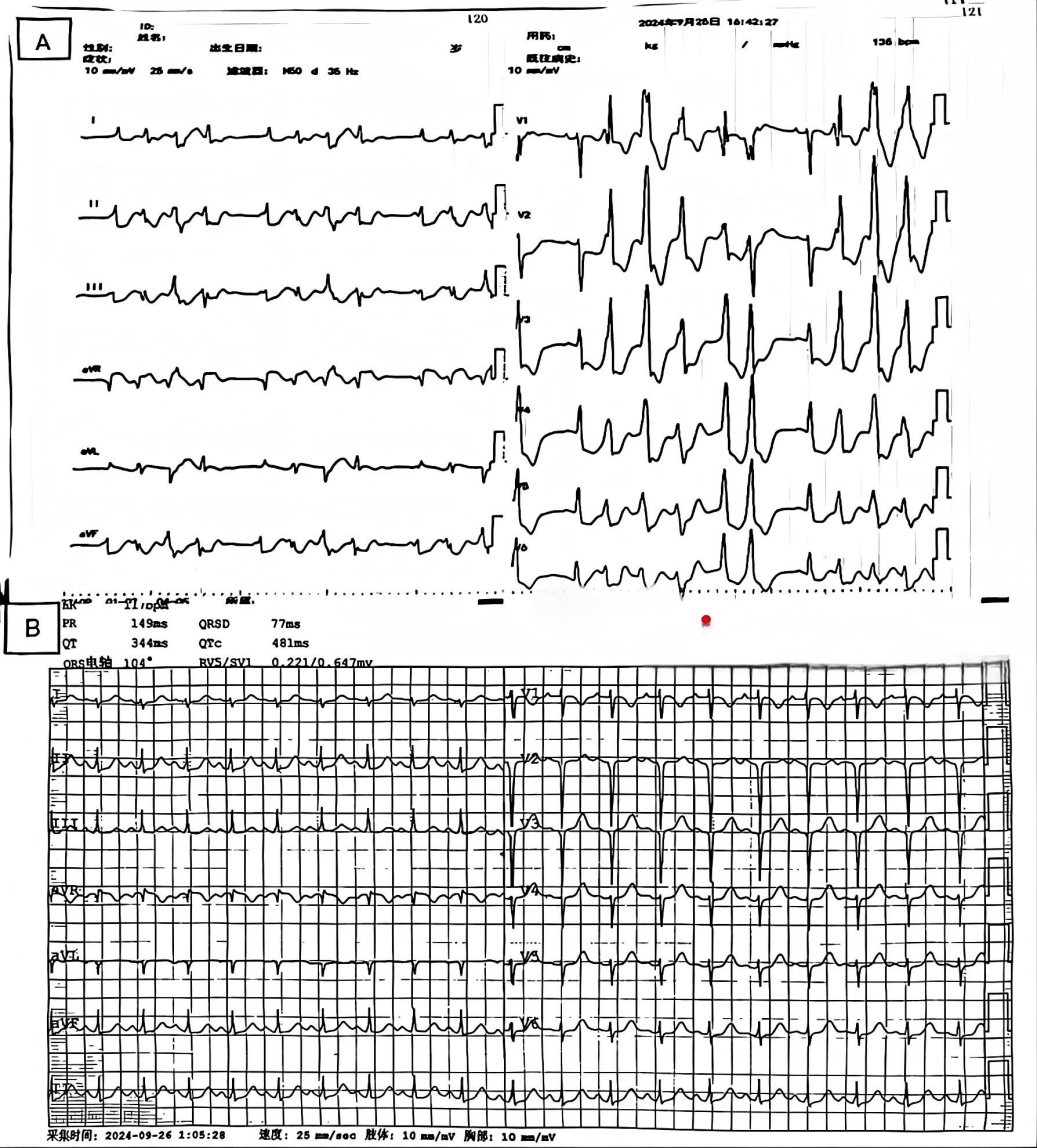
**(A)** Electrocardiogram (ECG) on admission, showing sinus rhythm combined with ectopic rhythm, non-sustained ventricular tachycardia (NSVT), and ST-T segment changes. **(B)** Repeat ECG after two hours showing evidence of anterior wall myocardial infarction.

The patient was an 11 year old child, admitted to the hospital due to “syncope” occurring 4 h prior. The patient experienced syncope during physical exertion and was admitted from the outpatient department under a diagnosis of “syncope”. Physical examination: T: 36.5 ℃, P: 118 beats/min, R: 29 breaths/min, Bp: 97/70 mmHg, SpO2 98%. The patient was conscious, with poor mental state, rapid breathing and slight difficulty in breathing. Pharynx is congested, no herpes, bilateral tonsils are grade I enlarged. No obvious dry or wet rales are heard in both lungs. Heart rate is rapid, rhythm is regular, heart sounds are low and dull. Abdominal examination and other systems showed no obvious positive signs. There was no sudden death, cardiomyopathy and other related family genetic history, and she suddenly fainted while running. The patient had no history of regular medication use before admission. The initial laboratory findings revealed markedly elevated cardiac enzyme levels, including: Myoglobin: 475.5 ng/mL (nr:0–65.8 ng/mL), LDH: 615.9 U/L (nr:120–250 U/L), CK: 4,334.4 U/L (nr:25–200 U/L), CK-MB: 328.00 ng/mL (nr:0–5 ng/mL), and cTnI: >100 ng/mL (nr:0–0.03 ng/mL). The attending physician initially suspected fulminant myocarditis. Electrocardiography (Figure 1A) revealed sinus rhythm with ectopic activity, non-sustained ventricular tachycardia, and ST-T segment abnormalities. Follow-up evaluation revealed persistent significant elevation of cardiac enzymes, while electrocardiographic progression demonstrated an anterior wall myocardial infarction pattern (Figure 1B).

**Figure 2:**
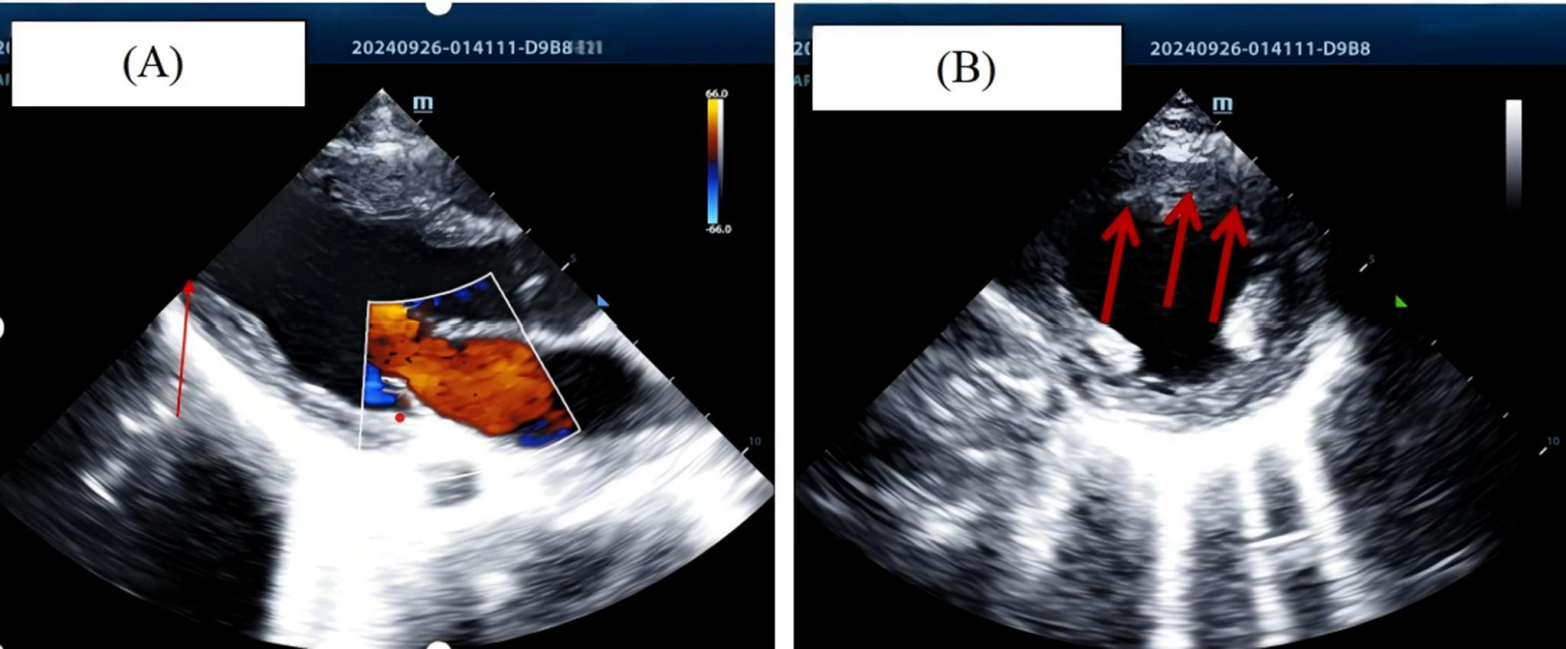
**(A)** short-axis view showing hypokinesis of the anterior left ventricular wall and apical segments. **(B)** Apical four-chamber view showing hypokinesis of the anterior left ventricular wall and apical segments.

Given the significant elevation in cardiac enzyme levels, urgent point-of-care echocardiography was performed. Echocardiographic findings (Figure 2) included hypokinesis of the mid-anterolateral left ventricular (LV) wall, the entire LV apex was visualized in parasternal long-axis, apical four-chamber, and short-axis views (Supplementary Movie S1–S3). Color Doppler flow imaging (CDFI) revealed mild mitral regurgitation. The patient's echocardiographic features were consistent with extensive anterior wall myocardial infarction. Upon retrospective review, it was found that the patient experienced a similar self-resolved episode (duration ∼2 min) 20 days prior, with no significant abnormalities detected during initial evaluation at a local hospital. The patient was suspected of having a coronary anomaly; based on the acute presentation and echocardiographic evidence of myocardial injury, anomalous coronary origin was strongly suspected.

**Figure 3:**
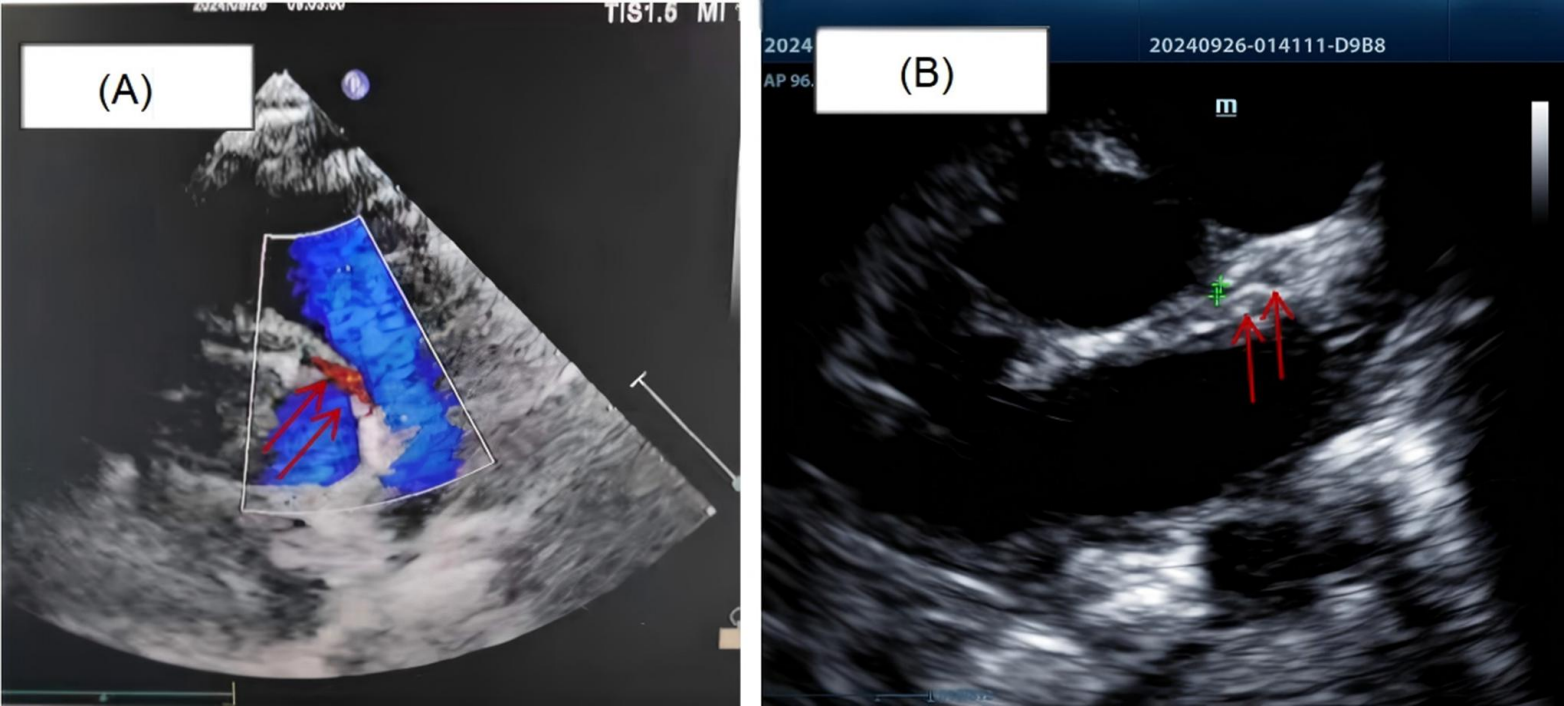
**(A)** the right coronary artery originates from the left coronary sinus, with an acutely angled ostium and an intramural course within the aortic wall. **(B)** The left coronary artery and its branches exhibit slender luminal caliber with reduced blood flow signals (marked by a green arrow).

Detailed coronary evaluation subsequently demonstrated that the right coronary artery (RCA) originated from the left coronary sinus with an acute angle of origin and an intramural aortic course (diameter: 2.3 mm). The left main coronary artery (LM) showed hypoplastic development (diameter: 1.3 mm) (Figure 3 and Supplementary Movie S4). Following interdisciplinary consultation and confirmation of anatomical abnormalities, the patient was transferred to a tertiary cardiovascular center for advanced management owing to critical clinical status, and family consent was obtained.

**Figure 4:**
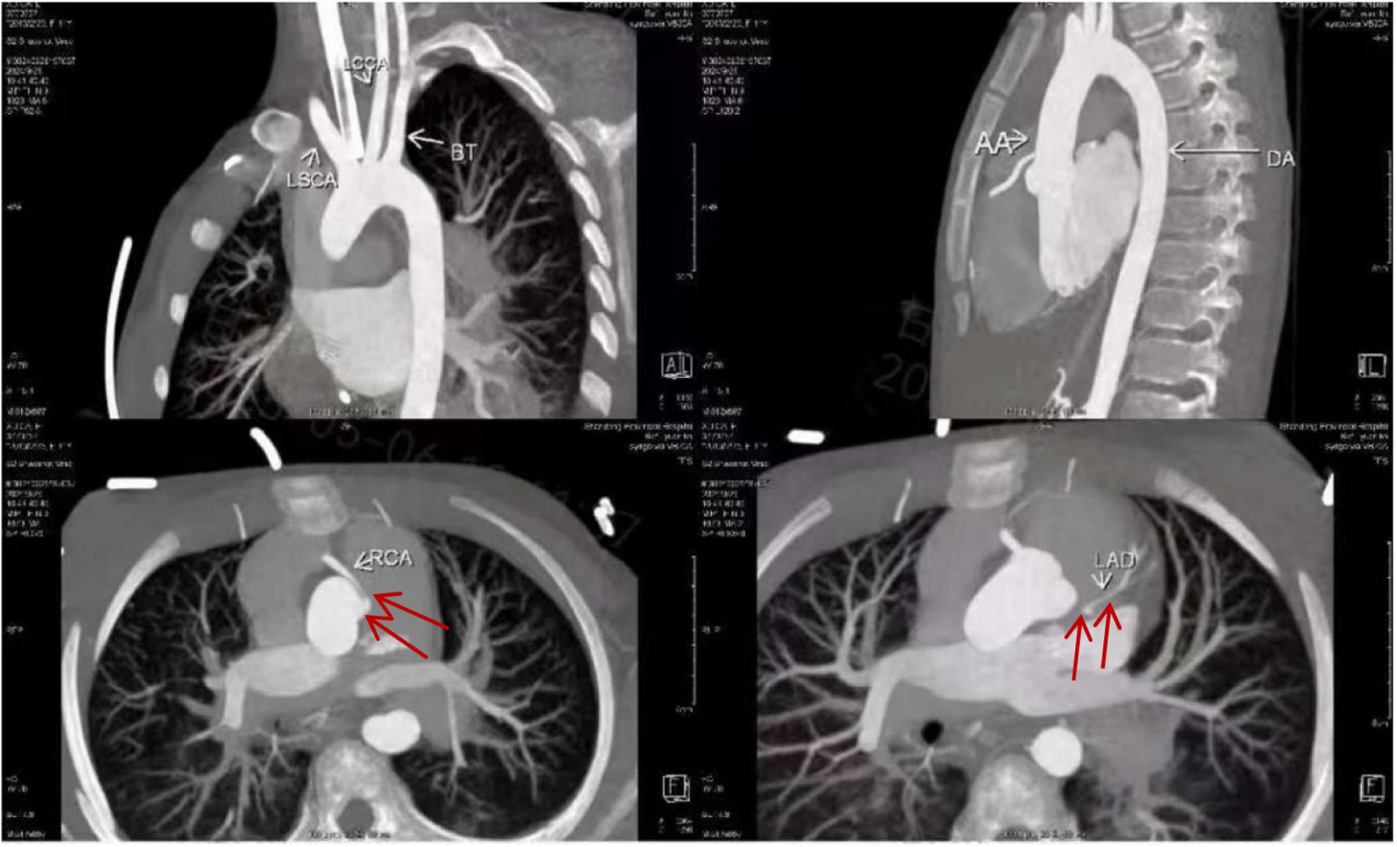
Coronary CTA shows the right coronary artery originates from the left coronary sinus and the left coronary artery and its branches exhibit slender luminal caliber (marked by a red arrow).

The follow-up coronary angiography results were completely consistent with the echocardiogram, showing that the right coronary artery originated abnormally from the left coronary sinus with significant dilation, consistent with a right-dominant circulation. The left anterior descending artery and the circumflex artery were visualized slightly later, and a thread-like blood flow was observed in the left main coronary artery (Figure 4). The patient then underwent right coronary artery ostial remodeling and left main coronary artery enlargement plasty first. However, weaning from cardiopulmonary bypass was difficult during the operation, and the left ventricular motion was poor. The surgical approach was finally changed to coronary artery plasty and coronary artery bypass grafting (10). The patient recovered well after surgery, and coronary perfusion was completely restored. During more than one year of follow-up, the child is currently in good condition and can live and move normall (Supplementary Figure S1).

## 3 Discussion

The patient presented with the classic manifestation of right anomalous coronary artery origin (R-AAOCA): sudden syncope during physical exertion. Transthoracic echocardiography confirmed the right coronary artery (RCA) originated from the left coronary sinus, with an acute ostial angle and an intramural aortic course—anatomical features that underpin symptom onset in R-AAOCA.

However, two key specificities of this case created a core discrepancy: ① concurrent left coronary artery (LCA) hypoplasia (diameter only 1.3 mm, markedly below the normal reference value for age-matched children); ② the patient was admitted with an anterior myocardial infarction (MI), a territory primarily perfused by the left anterior descending artery (LAD, a branch of the LCA) rather than the typical perfusion area of the anomalous RCA (posterior/inferior walls).

To address this discrepancy, two initial hypotheses were proposed, but both had limitations: (1) Dominant RCA with extensive perfusion: It was hypothesized that the anomalous RCA might be anatomically dominant, supplying the LAD territory via collaterals, but no evidence of robust collateral vessels was identified in this case; (2) Isolated LCA hypoplasia-induced ischemia: This hypothesis attributed the anterior MI solely to congenital LCA hypoplasia, but it failed to explain the patient's R-AAOCA-related exertional syncope. Thus, a more comprehensive and clinically congruent hypothesis is the synergistic ischemic effect of R-AAOCA and LCA hypoplasia: ① The intramural course of R-AAOCA is the core anatomical basis—during physical exertion, ventricular systole increases aortic wall tension, which further exacerbates luminal compression of the intramural RCA segment and significantly reduces RCA blood flow. Although the RCA does not directly perfuse the anterior wall, this compromise impairs its partial compensatory role for LCA-perfused territories at rest; ② Congenital LCA hypoplasia (diameter only 1.3 mm, markedly below the normal reference value for age-matched children (11). Results in inherent baseline hypoperfusion; ③ The patient developed symptoms during strenuous activity, which caused a sharp surge in myocardial metabolic demand. At this point, the compressed RCA could not increase blood supply via compensation, and the hypoplastic LCA was unable to meet the elevated oxygen requirements. This “dual hypoperfusion” synergistic effect led to anterior myocardial perfusion being far from sufficient to match metabolic needs, ultimately triggering ischemic necrosis and forming an anterior MI.

In this case, myocarditis was initially suspected due to exertional syncope, markedly elevated cardiac enzymes, and electrocardiographic ST-T abnormalities, requiring differentiation from primary arrhythmic causes and cardiomyopathy: myocarditis is centered on myocardial inflammation and typically lacks coronary structural anomalies, but this patient was confirmed by imaging to have an anomalous right coronary artery origin with left main hypoplasia, and electrocardiography showed an acute anterior myocardial infarction pattern, with myocardial injury caused by coronary ischemia inconsistent with an inflammatory mechanism; primary arrhythmias require a “structurally normal heart,” while this patient had definite coronary and echocardiographic structural abnormalities, and elevated cardiac enzymes indicated myocardial necrosis rather than isolated electrical disturbances; cardiomyopathy is characterized by intrinsic myocardial structural and functional abnormalities, but the left ventricular wall hypokinesis in this case was secondary to ischemia, with normal ventricular function restored after coronary revascularization, ruling out primary myocardial pathology. Ultimately, AAOCA combined with left coronary artery hypoplasia was confirmed as the definitive etiology of the patient's symptoms. It is important to clarify that isolated LCA hypoplasia can theoretically cause myocardial ischemia independently: its diameter is far below normal, resulting in inherent baseline hypoperfusion. During exertion, myocardial metabolic demand surges, and the vessel cannot compensate, potentially triggering ischemia, myocardial infarction, or sudden cardiac death (12). In a study, Clara Fiorentini and colleagues reported a case of sudden cardiac death caused by left coronary artery malformation, noting that isolated left coronary artery abnormalities are associated with sudden cardiac death (13). However, the critical difference in this case is that multimodal imaging and coronary angiography have clearly confirmed a combined anatomical anomaly of “R-AAOCA + LCA hypoplasia”, rather than isolated LCA hypoplasia. The core distinction between the two lies in the presence of objective anatomical evidence for R-AAOCA: “isolated LCA hypoplasia” is merely a theoretical speculation without supporting anatomical proof, while the “combined lesion” in this case is corroborated by cross-validation from multiple imaging modalities and intraoperative findings, forming a complete evidence chain.

Early diagnosis is a prerequisite for reducing the risk of sudden death in pediatric patients with R-AAOCA (14, 15). Conventional diagnostic modalities, such as coronary computed tomography angiography and invasive coronary angiography, remain the gold standard for this condition, as they enable definitive visualization of the coronary origin and course (16, 17). However, due to their invasive nature and high cost, these techniques are not routinely employed as first-line diagnostic tools for the evaluation of syncope (18).

Direct signs on echocardiography include abnormal coronary ostial location and course, whereas indirect signs may manifest as myocardial ischemia in the corresponding coronary perfusion territories. Nevertheless, the detection rate of direct echocardiographic signs remains low (19, 20), with the existing literature reporting suboptimal diagnostic accuracy for anomalous coronary origins, particularly during initial examinations. This limitation is primarily attributed to artifacts, limited spatial resolution, and insufficient awareness of anomalies amongst clinicians. Consequently, R-AAOCA is rarely diagnosed on initial echocardiography, and is predominantly identified by coronary CTA or angiography (21, 22). To clarify the roles of different imaging modalities in addressing these limitations and optimizing the diagnostic pathway, Table 1 systematically presents the “echocardiography → coronary CTA → invasive angiography” sequence, key findings, and specific contributions to diagnosis and surgical planning (Table 1).

**Table 1:** Echo → CT → angiography function.

Examination modality	Core findings	Diagnostic/surgical planning value
Transthoracic Echocardiography (TTE)	Anomalous origin of the right coronary artery (arising from the left coronary cusp); Slender main trunk and branches of the left coronary artery (LCA); Hypokinesis of the anterior wall of the left ventricle; - Doppler echocardiography shows increased systolic blood flow velocity in the intramural segment of R-AAOCA (peak 2.8 m/s, diastolic 1.2 m/s)	First-line screening tool: rapidly indicates anatomical abnormalities and myocardial dysfunction, and guides subsequent advanced examinations.
Coronary Computed Tomography Angiography (CCTA)	Clarifies R-AAOCA anatomy: origin from the left coronary cusp, intramural course for 12 mm before piercing the myocardium; LCA diameter 2.1 mm (normal reference value for peers: 4.8 ± 0.6 mm); No coronary artery calcification or thrombosis; Myocardial edema in the anterior wall of the left ventricle (positive late gadolinium enhancement)	Gold standard (referring to AHA guidelines): accurately evaluates vascular origin, course, and lumen morphology, providing anatomical basis for the selection of surgical approach.
Invasive Coronary Angiography	Verifies CCTA findings; R-AAOCA ostium is slit-like (diameter 1.5 mm); TIMI grade Ⅱ blood flow in the left anterior descending artery (LAD) and left circumflex artery (LCX); Exercise stress test (dobutamine) shows 70% systolic luminal stenosis of R-AAOCA	Dynamically assesses hemodynamics, confirms the degree of obstruction, and rules out other coronary artery lesions.

Recent studies, such as those by Bianco et al., have indicated that standardized echocardiographic protocols may improve the diagnostic sensitivity of R-AAOCA (23). Accordingly, in recent years transthoracic echocardiography (TTE) has gained increasing recognition as a valuable tool for R-AAOCA detection (24, 25).

As such, prioritizing comprehensive coronary artery evaluation combined with high-quality 2D and CDFI modalities is critical for enhancing the diagnostic accuracy for coronary anomalies, minimizing missed diagnoses and misdiagnoses, and providing more reliable etiological insights for patients presenting with syncope. As such, TTE should be established as a pivotal screening tool for R-AAOCA.

## Data Availability

The original contributions presented in the study are included in the article/Supplementary Material, further inquiries can be directed to the corresponding authors.

## Supplementary material

Supplementary Figure S1 Timeline figure that clearly presents key nodes including symptom onset (first episode 20 days prior to presentation, recurrent syncope 4 h before admission), examinations (imaging, ECG, etc.), surgery, and follow-up, intuitively illustrating the temporal sequence of the case.

Supplementary Movie S1 Long-axis view showing hypokinesis of the anterior left ventricular wall and apical segments.

Supplementary Movie S2 Apical four-chamber view showing hypokinesis of the anterior left ventricular wall and apical segments.

Supplementary Movie S3 Short-axis view showing hypokinesis of the anterior left ventricular wall and apical segments.

Supplementary Movie S4 Dynamic display the right coronary artery originates from the left coronary sinus and the left coronary artery and its branches exhibit slender luminal caliber.
