# Supplementary figures and images for: Case Report: Syncope in an 11-year-old girl induced by anomalous aortic origin of the coronary artery, initially diagnosed via echocardiography
Source: Front Cardiovasc Med. 2026 Feb 18;13:1632958. doi: 10.3389/fcvm.2026.1632958 (PMC12957173; doi:10.3389/fcvm.2026.1632958)

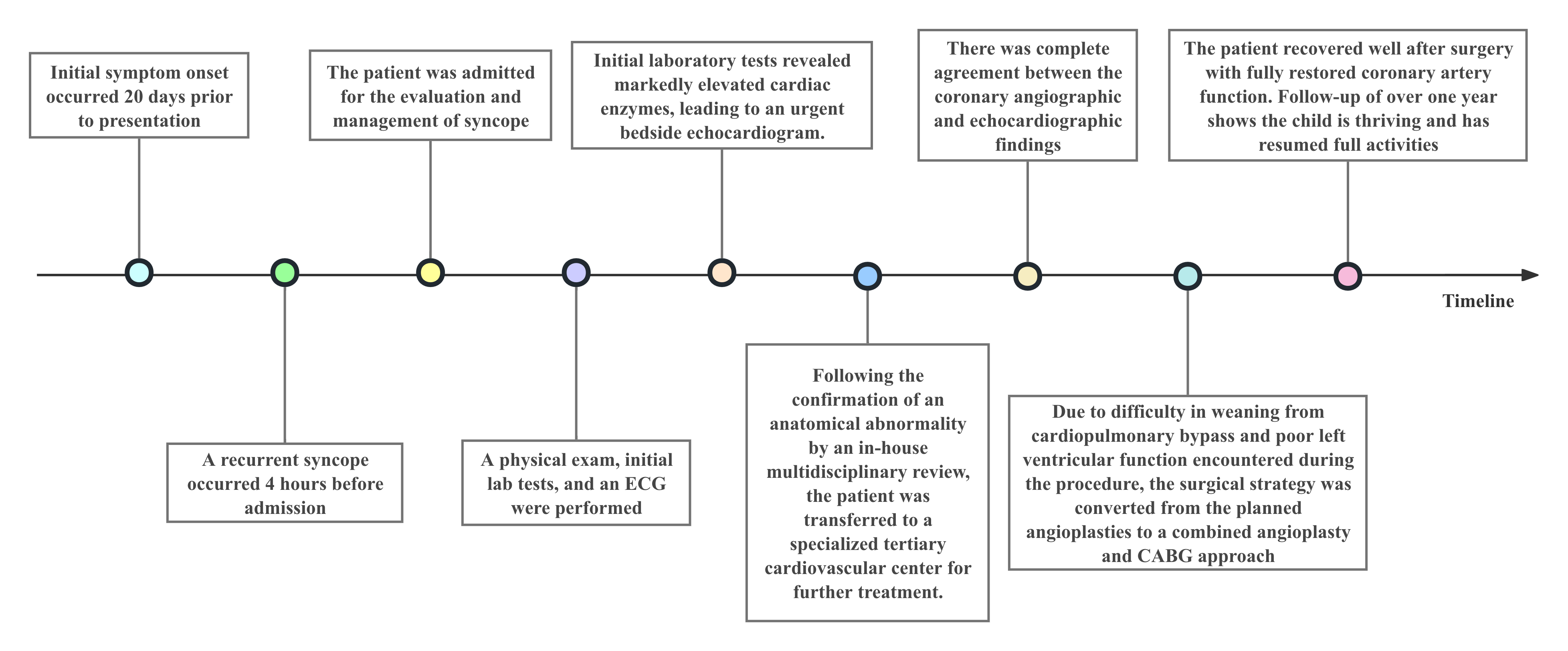

Supplement: Supplementary Figure S1 — Timeline figure that clearly presents key nodes including symptom onset (first episode 20 days prior to presentation, recurrent syncope 4 h before admission), examinations (imaging, ECG, etc.), surgery, and follow-up, intuitively illustrating the temporal sequence of the case. [file Image1.tif]
